# Physiological and histopathological responses of *Pangasianodon hypophthalmus* to ammonia exposure at varying thermal regimes

**DOI:** 10.1038/s41598-026-58905-0

**Published:** 2026-06-28

**Authors:** Rehab A. Abd-elaziz, Safaa H. Aboolo, Mustafa Shukry, Mahmoud S. Gewaily, Hany M.R. Abdel-Latif

**Affiliations:** 1 Fish Diseases Department, Alexandria Provincial Lab, Animal Health Research Institute (AHRI), Agriculture Research Center (ARC), Giza, Egypt; 2 Pathology Department, Agriculture Research Center (ARC), Animal Health Research Institute (AHRI), Giza, Egypt; 3 Department of Physiology, Faculty of Veterinary Medicine, Kafrelsheikh University, Kafrelsheikh, 33516 Egypt; 4 Department of Biomedical Sciences, College of Veterinary Medicine, King Faisal University, P.O. Box 400, Al-Ahsa, 31982 Saudi Arabia; 5 Department of Anatomy and Embryology, Faculty of Veterinary Medicine, Kafrelsheikh University, Kafrelsheikh, 33516 Egypt; 6 Department of Poultry and Fish Diseases, Faculty of Veterinary Medicine, Alexandria University, Alexandria, 22758 Egypt

**Keywords:** Ammonia, Oxidative stress, Lipid peroxidation, Pangasius, Biochemistry, Biomarkers, Diseases, Environmental sciences, Physiology, Zoology

## Abstract

This study evaluated the effects of ammonia exposure on the physiological responses, vitality, and histopathology of *Pangasianodon hypophthalmus* at two temperatures. A 2 × 2 factorial design was applied using two total ammonia nitrogen (TAN) levels (0 and 10 mg/L) and two temperature regimes (28 °C and 32 °C) over a 4-week period. At the end of the experiment, clinical signs, histopathology, hematology, stress indicators (glucose and cortisol), liver function enzymes, and oxidative stress markers (malondialdehyde levels and antioxidant enzymes) were assessed. Fish exposed to 10 mg/L TAN at 32 °C exhibited severe clinical signs, including dermal erosion, muscle necrosis, and respiratory distress. Ammonia exposure at 32 °C significantly reduced red blood cell (RBC) counts, hemoglobin, and hematocrit. Furthermore, elevated temperatures exacerbated ammonia-induced stress, evidenced by significant increases in cortisol, blood glucose, and malondialdehyde levels, alongside altered liver and antioxidant enzyme activities. Histopathological analysis confirmed significant damage to the gill filaments, hepatopancreas, and renal tissues, with severity increasing alongside water temperature. These results indicate a synergistic effect between ammonia exposure and water temperatures, where higher temperatures reduce the threshold for ammonia tolerance, triggering respiratory distress, systemic oxidative stress, and metabolic failure. These findings underscore the critical need for strict water quality management in tropical aquaculture, particularly in regions like Egypt, as rising global temperatures due to climate change may transform currently sub-lethal ammonia levels into potent lethal stressor for *P. hypophthalmus*. However, the study is limited by controlled laboratory conditions and relatively short experimental duration, suggesting the need for long-term investigations.

## Introduction

In the aquatic ecosystems, ammonia is ubiquitous and produced from several sources such as excessive use of organic fertilizers, and the presence of decayed plants and animals. However, the high level of ammonia can be attributed by agricultural and industrial waste, which appears as a result of the decomposition of the nitrogenous organic materials^1^. Research elucidated that exposure to high ammonia concentrations can provoke a range of deleterious consequences in the exposed aquatic animals. Acute ammonia exposure can also induce respiratory distress manifested by increased gill ventilation, impaired respiratory function, loss of equilibrium, hyperexcitability, convulsions, and ionic balance failure^2^. In addition, ammonia toxicity can be manifested by growth depression, haemato-biochemical changes, worsened antioxidant defense mechanisms, immunosuppression, impaired metabolic functions, neurotoxicity, and osmoregulatory failure^3,4^. Ammonia also induces several physiological changes including high cortisol secretion and stimulation of glycogenesis, gluconeogenesis, protein catabolism^5^, and disruption of the ionic balance across the gills^6^. In general, ammonia in the aquatic ecosystem exists in two main forms: ionized ammonia form (NH^4+^) and unionized ammonia form (NH_3_), and both are considered as toxic to the exposed fish; however, NH_3_ is more toxic than NH^4+^, because of its potential ability to diffuse easily through the epithelial membranes^7^. Ammonia toxicity is affected by other environmental factors such as water temperature, pH, and salinity^8^.

Water temperature is considered as one of the most critical factors that affect the degree of ammonia toxicity in fish due to it affects the bioavailability and forms of ammonia^9^. In general, the elevated water temperature can enhance ammonia toxicity because it can influence the ammonia diffusion rate through gill membranes and activate chemical reactions^10,11^. Climate changes and global warming influence the widespread high-temperature waves that deleteriously affect all human activities including agriculture and aquaculture^12^. The high temperature induces thermal stress in aquatic animals leading to metabolic dysfunctions and high mortalities in aquaculture^13^. High water temperatures can induce several physiological disturbances, oxidative stress and immune suppression of the exposed fish^14,15^.

Striped catfish, *P. hypophthalmus* is a tropical stenothermal species with a relatively narrow ideal thermal window, typically cited between 26 °C and 30 °C^16^. While it can survive short-term exposure to temperatures as low as 22 °C or as high as 39 °C, prolonged deviations from the optimum range significantly impair metabolic growth rates and immune functions of the exposed fish. Regarding the nitrogenous wastes, this species is noticed for its relative hardiness compared to other teleosts; however, chronic ammonia exposure remains a critical stressor. Research studies indicate that the median lethal concentration (LC50) for TAN over 96 h often exceeds 25 mg/L^17^, but sub-lethal physiological disturbances such as gill histological damage and oxidative stress are observed at much lower concentrations, typically starting around 1.0 to 2.0 mg/L of NH_3_.

Several studies have indeed shown that higher water temperatures can increase the toxicity of ammonia to aquatic organisms. For example, it was found that dual exposure of ammonia and thermal stress induced oxidative stress dysfunction and immunity depression in Nile tilapia (*Oreochromis niloticus*)^18^. No previously published studies were carried out to explore the negative consequences of ammonia exposure on the performance, health, and vitality of *P. hypophthalmus* reared at ambient water temperatures. As a result, the present research was formulated to analyze the influences of two levels of ambient temperature (28–32 °C) and two TAN levels (0 and 10 mg/L) on the overall health, physiological responses, histopathological alterations and vitality of *P. hypophthalmus*.

## Materials and methods

### Experimental animals and acclimation conditions

*P. hypophthalmus*, were purchased from a private fish farm at Alexandria-Desert Road (Dr. Belal W. Allam Hatchery) and then were transferred to well-aerated plastic tanks to the Fish Diseases Department, Alexandria Provincial Lab, Animal Health Research Institute, Alexandria, Egypt, where the fish experiments were carried out.

Fish were left in the tanks (250 L water capacity) for 15 days to be acclimatized to the laboratory conditions. During this period, fish were fed twice daily (3% of their body weight) on a well-balanced commercially purchased diet (32% crude protein and 6% fat) (Aller Aqua Co., Egypt). Fish were reared in dechlorinated filtered tap water with 0‰ salinity, 28.0 °C temperature, 7.50 ± 0.20 pH value, and 6.5 ± 0.50 mg/L dissolved oxygen (DO). After acclimatization, 120 fish (mean length 12.30 ± 1.10 cm, and mean weight 18.25 ± 2.4 g) were randomly selected for the present study. Fish were distributed into 12 glass aquaria (100 cm × 90 cm × 70 cm), each with a 90-L water capacity, and each group contained 30 animals, and each aquarium contained 10 individuals. Fish were reared in these aquaria throughout the experimental period. About 1/3 of the water per aquarium has been daily changed. Each aquarium had 2 air stones connected with an aerator to maintain suitable aeration. Fish were maintained on a 24-h dark cycle and constant conditions.

### Experimental setup and husbandry details

The experiment was carried out on a 2 × 2 factorial design with two levels of ammonia (0 and 10 mg/L) and two ambient water temperatures (28 and 32 °C) in triplicates.

#### Temperature control clarification

To prevent thermal shock, the water temperature increased incrementally by 1 °C per day until it reached the target experimental temperature of 32 °C. This temperature was maintained for a 24-hour stabilization period, during which the fish exhibited normal feeding behavior and no signs of stress. For the experimental treatments, temperatures were maintained at 28 °C and 32 °C using submerged thermostatic heaters to ensure consistency regardless of ambient room temperature. These values align with the optimal growth range for *P. hypophthalmus* (26–32 °C) as established by Nguyen^16^. By maintaining the treatments within these established thermal levels, the potential for confounding effects resulting from thermal stress or heat-induced metabolic exhaustion was minimized, ensuring that the observed responses were representative of standard physiological conditions.

####  Ammonia exposure rationale

Ammonium chloride (NH_4_Cl) was purchased from El-Gomhouria Co. (Alexandria, Egypt). Every day, NH_4_Cl solution was freshly prepared every day by mixing with dechlorinated filtered tap water and adjusted to 10 mg total ammonia nitrogen (TAN) per liter. A TAN dose of 10 mg/L was selected as a sub-lethal ammonia stressor according to the results published in the same fish species (*P. hypophthalmus*) by Truc et al^17^.. Those authors found that the LC50-96 h (50% mortality within 96 h) of unionized ammonia (NH_3_) was 3.52–3.97 mg/L (equivalent TAN of ~ 60.6 mg/L) and the safe NH_3_ concentration was ~ 0.20 mg/L (equivalent TAN of 3 mg/L) with no significant impacts on survival accompanied with minimal stress. In the present study, the selection of 10 mg/L TAN represents an artificially induced stress condition designed to simulate a worst-case scenario often encountered in the intensively high-density aquaculture, where filtration systems may fail or water exchange is limited. This dose was chosen to trigger a robust antioxidant and immunological response, allowing us to observe the fish’s capacity for homeostasis under significant environmental pressure.

#### Experimental setup

The experiment continued for 4 weeks, and fish were grouped in triplicate into 4 main groups as follows: 

**Table:** 

Groups	Treatment	No. of reps
Group 1	Fish were reared at 28 °C without ammonia exposure (0 mg/L)	3
Group 2	Fish were reared at 28 °C and exposed to TAN (10 mg/L)	3
Group 3	Fish were reared at 32 °C without ammonia exposure (0 mg/L)	3
Group 4	Fish were reared at 32 °C and exposed to TAN (10 mg/L)	3

TAN values vary significantly with temperature and pH values. During the experiment, continuous monitoring and operational stability have been carefully followed to ensure neutral pH is maintained (pH 8.0), thus validating the consistency of ammonia exposure concentration across all test replicates. In the present experiment, the calculated NH_3_ values were 0.6829 mg/L at 28 °C and 0.9112 mg/L at 32 °C. In addition, the settled fish waste was regularly daily siphoned along with 35% of the water from each aquarium, which was replaced by clean and well-aerated water containing the same TAN exposure dose according to the experimental design.

### Clinical examination

Clinical examination procedures were carried out on the freshly dead and moribund fish to evaluate the abnormal external signs and necropsy findings to record any postmortem (PM) lesions^19–21^. Respiratory distress was assessed qualitatively based on opercular movement rate, gasping behavior, and loss of equilibrium.

### Sampling: types and procedures

After the experiment ended, six fish from each group were anesthetized with 50 mg/L clove oil (El-Gomhouria Co., Alexandria, Egypt) to reduce the handling stress. Samples and sampling procedures were carried out according to our published study^22^. Fish were stared 24 h prior to blood sampling. In brief, two sets of blood samples were drawn from caudal veins without sacrificing the fish; the 1 st set was used on the same sampling day using an anticoagulant for the determination of the hematological parameters, while the second set of blood samples was collected and transferred to sterile, labeled Eppendorf tubes to separate the serum. The samples were left for an hour at room temperature in a vertical position to allow the serum to separate, then centrifuged at 3500 × g for 12 min. The serum was then collected with a sterile micropipette and stored at −20 °C for later analysis of serum biochemical indices according to our previously published research^23^. All centrifugation steps were performed at 4 °C using a refrigerated centrifuge.

Following the blood collection, fish were necropsied under complete aseptic conditions to sample organs and tissues. Euthanasia of all fish samples were carried out using an overdose (120 mg L^[-1^ of benzocaine. Livers, dorsal muscle, posterior kidney, and gills were collected, rinsed with a cold, sterile PBS solution, and then preserved in 10% buffered formalin for histopathological studies. Additional liver samples were also collected, kept on ice, and then stored at −20 °C for the determination of lipid peroxidation and oxidative stress indicators.

### Histopathological alterations

Examination of the histopathological alterations that occurred in gills, hepatopancreas, posterior kidney, and muscular tissues of different experimental groups were adopted according to procedures described in Bancroft and Gamble^24^. In brief, the tissue samples were fixed in 10% neutral buffered formalin for two days, dehydrated in ascending ethyl alcohol concentrations, and impregnated in paraffin. Then, several 5 μm-thick sections were obtained with a Leica rotatory microtome (RM 20352035; Leica Microsystems, Germany) and stained with H & E. The stained tissue sections were examined using a light microscope (BX50/BXFLA, Olympus, Tokyo, Japan).

### Hematological picture

Red blood cell (RBCs) count, leucocyte count (WBCs), and differential leukocyte counts were performed on the blood samples according to the methods described by Dacie and Lewis^25^, and cells were counted in an improved Neubauer hemocytometer. The standard micro-hematocrit method was used to measure Hematocrit (Htc) values^26^ using Micro-haematocrit Centrifuge. The hemoglobin (Hb) content was determined by the acid haematin method using Sahli’s haemoglobinometer^27^.

### Stress indicators

According to the manufacturer’s instructions, blood glucose (mg/dL) was measured using commercial kits (Glucose-HK Ref 1001200; Spinreact Co., Girona, Spain), adapted for 96-well microtiter plates. Cortisol (ng/dL) levels were determined using commercially purchased ELISA kits (Fish Cortisol ELISA Kit, CSB-E08487f, Cusabio Biotech Co., Ltd., Wuhan, China).

### Liver function enzymes

According to the manufacturer’s instructions, the liver function enzymes were estimated using commercial kits (Biodiagnostic Co., Giza, Egypt). Serum alanine transaminase (ALT) and aspartate transaminase (AST) activities were determined colorimetrically according to Reitman and Frankel^28^. Alkaline phosphatase (ALP) activity was measured according to Belfield and Goldberg^29^.

### Lipid peroxidation and enzymatic oxidative stress biomarkers

Liver samples that were stored at −20 °C were transferred to an ice-water bath to be melted, and 0.1 g of the tissues were homogenized with 0.9 mL of PBS solution (pH ~ 7.4) in a Teflon-coated mechanical homogenizer. Tissue homogenates were then centrifuged at 3500 × g for 15 min, and the sediment was discarded, and the supernatant was taken for measurements. Following the manufacturer’s instructions, the enzymatic oxidative stress biomarkers parameters, including catalase (CAT), superoxide dismutase (SOD), and glutathione peroxidase (GPx) activities, were determined using fish-specific commercial kits (Fish Catalase (CAT) ELISA kit (CSB-E15928Fh), Fish Superoxide Dismutase (SOD) ELISA kit (CSB-E15929Fh), and Fish Glutathione Peroxidase (GSH-PX) ELISA kit (CSB-E15930Fh). These kits were purchased from Cusabio Biotech Co., Ltd., Wuhan, China. Glutathione-S-transferase (GST) was determined using the GSTA1 ELISA kit: Fish Glutathione S Transferase Alpha 1 ELISA Kit (MyBioSource, Inc., San Diego, CA, USA). The malondialdehyde (MDA) concentrations in liver samples were also measured using commercially purchased kits (Lipid peroxide, MDA) from Biodiagnostic Co., Giza, Egypt.

### Statistical evaluation

Shapiro-Wilk test was used to check the normality before statistical analysis. The obtained results were subjected to a two-way ANOVA to explore the effects of ammonia and temperatures as the two factors were simultaneously tested. The differences between means were examined using Duncan’s multiple range test as a post hoc test at the 5% probability level (*P* < 0.05). All analyses were conducted using SPSS version 20 (SPSS, Richmond, VA, USA). To ensure clear biological interpretation, mean separation letters were assigned based on Tukey’s Honest Significant Difference (HSD) test across all treatments, to decompose the interaction and evaluate the effect of ammonia within each temperature level, as well as the effect of temperature within each ammonia concentration.

## Results

### Clinical signs and PM lesions

**Fig. 1:**
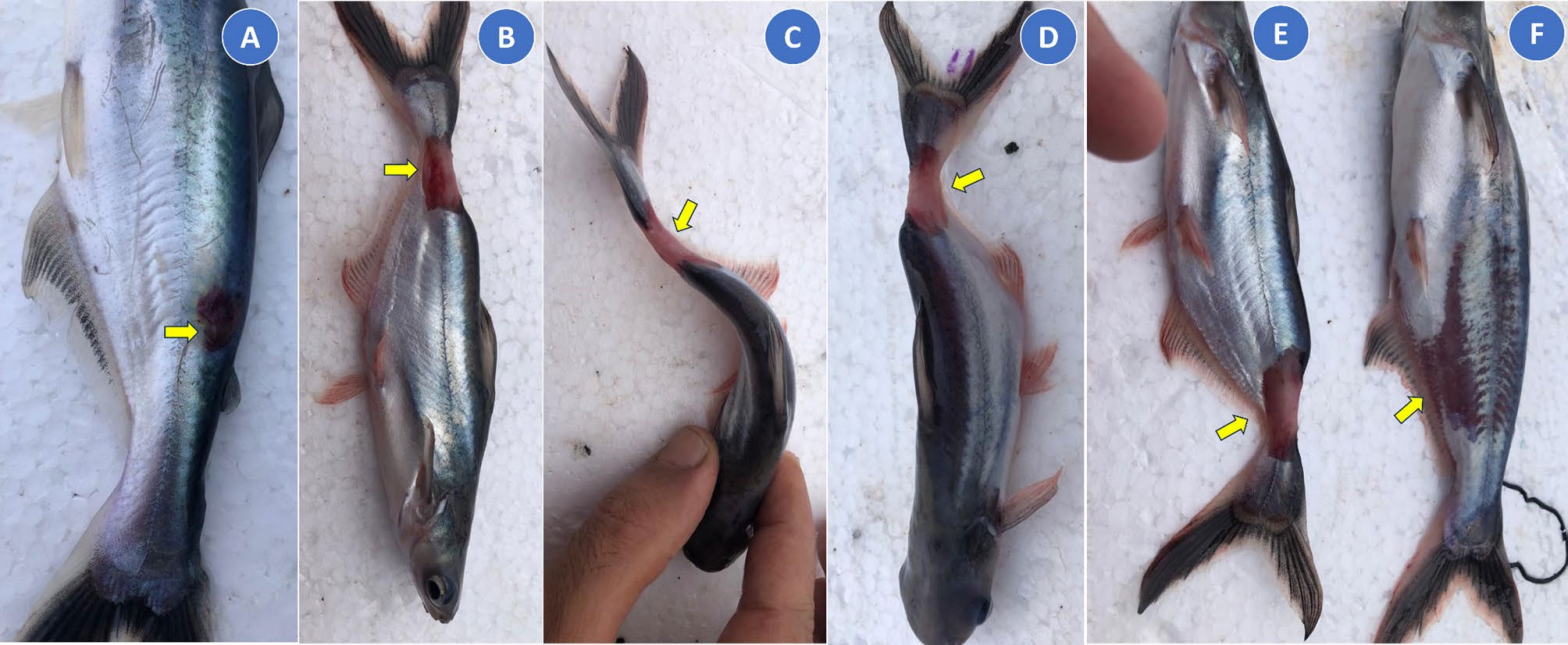
The clinical picture of a group of *P. hypophthalmus* that was exposed to sub-lethal ammonia and reared at a water temperature of 28 °C, showing dermal erosions (**A**), necrosis, and sloughing of the musculature at the caudal peduncle (**B**, **C**, and **D**). Another fish group was exposed to sub-lethal ammonia and reared at a water temperature of 32 °C, showing sloughing caudal peduncle (**E**) and extreme muscle necrosis and ulcerations (**F**).

**Fig. 2:**
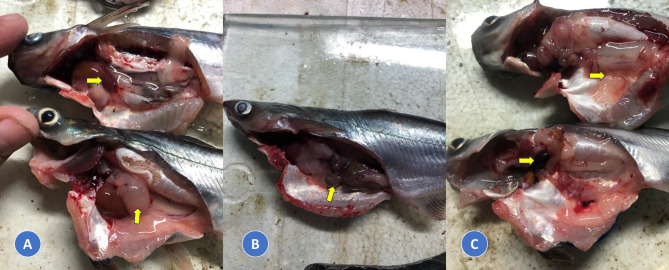
The PM findings of a group of *P. hypophthalmus* that was exposed to sub-lethal ammonia and reared at a water temperature of 28 °C, showing an engorged gall bladder and congested liver (**A**) and congested intestinal tract (**B**). Another fish group was exposed to sub-lethal ammonia and reared at a water temperature of 32 °C, showing exophthalmia, serous ascitic abdominal fluid and a congested spleen (**C**).

At the end of the experiment, *P. hypophthalmus* reared at 28 °C and 32 °C did not exhibit any abnormal clinical signs or PM lesions. However, fish exposed to sub-lethal ammonia and reared at 28 °C exhibited slight respiratory and nervous manifestations and showed dermal erosions (Fig. 1A), necrosis, and sloughing of the musculature at the caudal peduncle (Fig. 1B, C, *and D*). However, the fish group that was exposed to sub-lethal ammonia and reared at 32 °C ceased feeding, sever respiratory distress and nervous manifestations, and showed high degree of muscular sloughing at the caudal peduncle (Fig. 1E) and extreme muscle necrosis and ulcerations in few cases (Fig. 1F). The necropsy findings revealed that *P. hypophthalmus* group that was exposed to sub-lethal ammonia and reared at a water temperature of 28 °C showed engorged gall bladder and congested liver (Fig. 2A) and congested intestinal tract (Fig. 2B). Of interest, the fish group that was exposed to sub-lethal ammonia and reared at a water temperature of 32 °C showed serous ascitic abdominal fluid and congested spleen (Fig. 2C).

### Histopathological findings

#### Gills

**Fig. 3:**
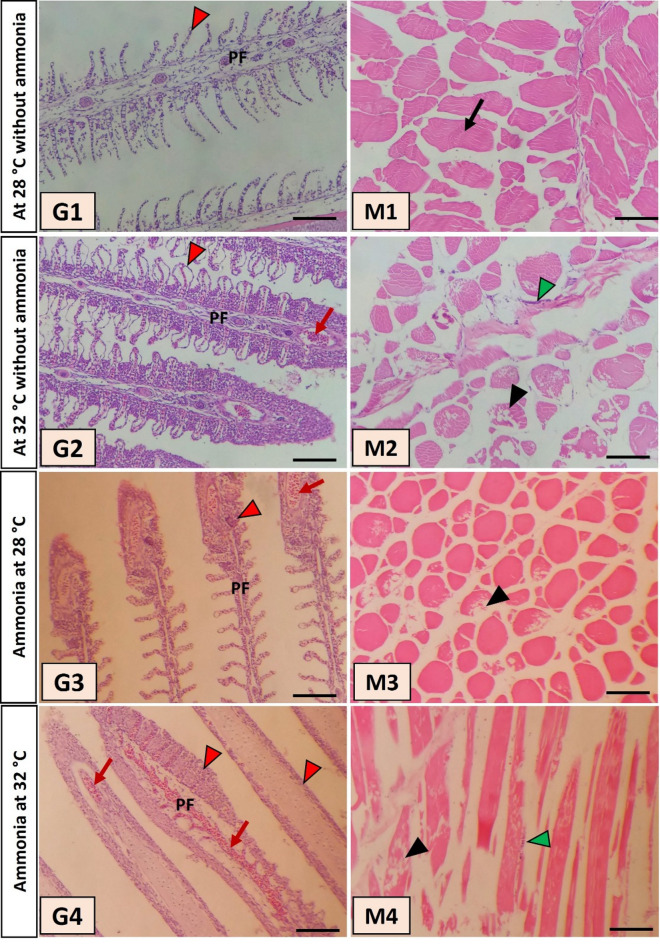
Photomicrographs of the gill tissues of* P. hypophthalmus* in groups (G1 and G2) that were reared at different water temperatures (28 °C and 32 °C) without ammonia exposure and groups (G3 and G4) that were reared at different water temperatures (28 °C and 32 °C) and exposed to sub-lethal ammonia exposure. G1-G4; primary filaments (PF) and secondary filaments (red arrowhead), blood vessels (red arrow). Photomicrographs of the muscles in groups (M1 and M2) that were reared at different water temperatures (28 °C and 32 °C) without ammonia exposure and groups (M3 and M4) that were reared at different water temperatures (28 °C and 32 °C) and exposed to sub-lethal ammonia. M1-4: Normal striation of the muscle fibers (black arrow), loss of Cohnheim appearance and Zenker’s necrosis (black arrowhead), and infiltration of the inflammatory cells (green arrowhead). Stain H&E. Scale bar: 100 µm.

The histopathology of the gills in the fish group reared at 28 °C without ammonia exposure showed that the gill filaments had centrally located cartilaginous bars with intact primary and secondary gill lamellae (Fig. 3, G1). However, fish reared at 32 °C without ammonia exposure revealed slight congestion of the primary filament blood vessels, deformed secondary lamellae with necrosis, and dilatation of the blood vessels (Fig. 3, G2). Fish reared at 28 °C with ammonia exposure triggered degeneration and necrosis of both primary and secondary filaments, inflammatory cell infiltration (Fig. 3, G3), while those reared at 32 °C, accompanied by ammonia exposure, triggered edema of primary filaments and sloughing of secondary filaments (Fig. 3, G4).

#### Musculature

The muscular tissue in the group reared at 28 °C without ammonia exposure (Fig. 3, M1) showed normal muscle fibers with the characteristic Cohnheim appearance, which appears as polygonal areas in the cross-sections of the striated muscle fibers. However, the fish group reared at 32 °C without ammonia exposure (Fig. 3, M2), and those exposed to sub-lethal ammonia and reared at 28 °C and 32 °C (Fig. 3, M3-M4), there was loss of the cohnheim appearance, condensation of the cytoplasm of myofibrils, and accompanied with Zenker’s necrosis with infiltration of the inflammatory cells.

#### Hepatopancreas

**Fig. 4:**
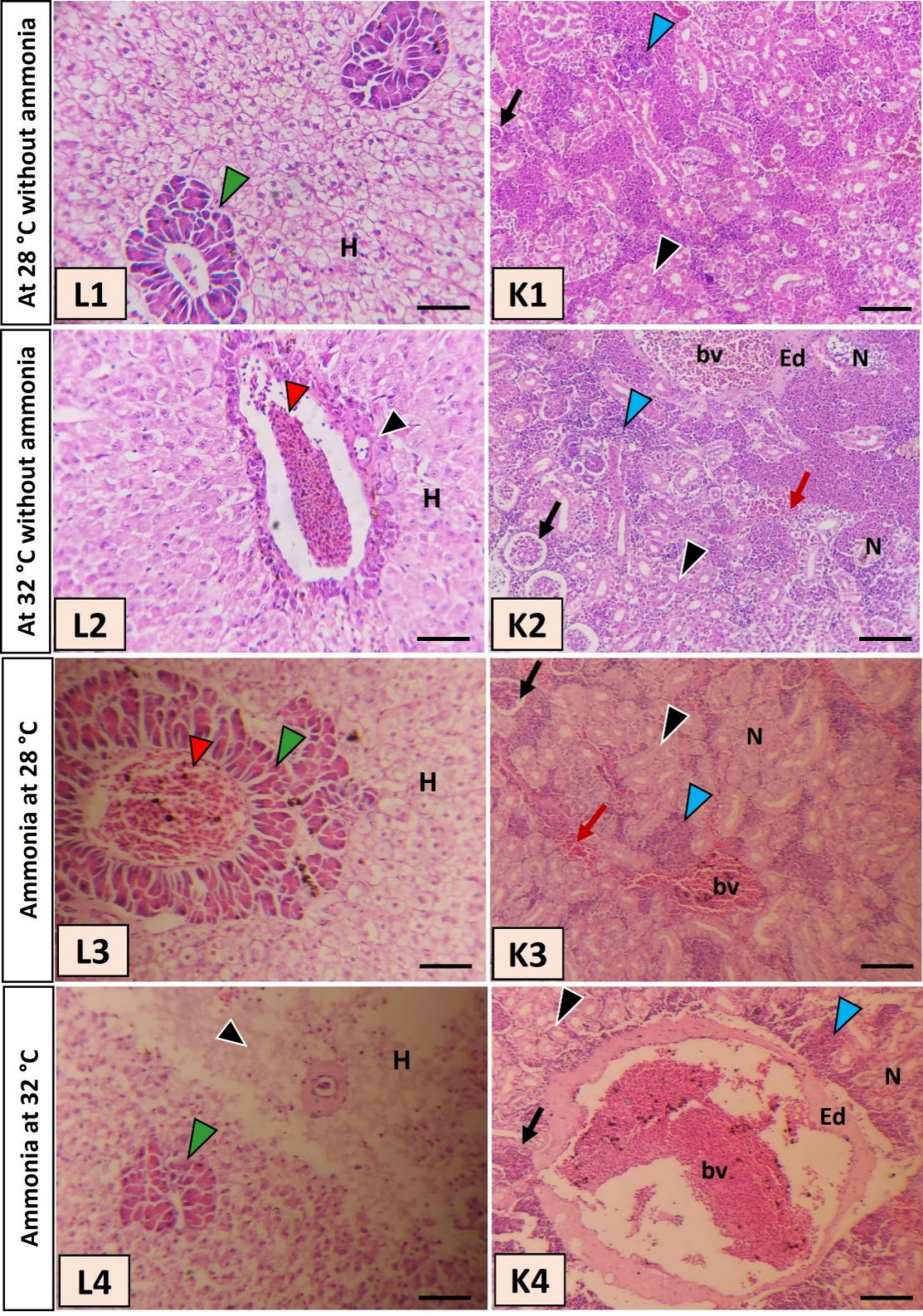
Photomicrographs of the hepatopancreas of *P. hypophthalmus* in groups (L1 and L2) that were reared at different water temperatures (28 and 32 °C) without ammonia exposure and groups (L3 and L4) that were reared at different water temperatures (28 and 32 °C) and exposed to sub-lethal ammonia exposure. L1-4; hepatic tissue (H) and pancreatic acinar cells (green arrowhead), dilatation and congestion of the blood vessels (red arrowhead) with necrosis of the hepatic cells (black arrowhead). Photomicrographs of the posterior kidney in groups (K1 and K2) that were reared at different water temperatures (28 and 32 °C) without ammonia exposure and groups (K3 and K4) that were reared at different water temperatures (28 °C and 32 °C) and exposed to sub-lethal ammonia exposure. K1-4; the bowman’s corpuscle (black arrow), interstitial infiltration of the inflammatory cells (blue arrowhead), necrosis (N) and edema (Ed), congestion of the blood vessels (bv), and extravasation of RBCs (red arrow). Stain H&E. Scale bar: 50 μm.

The hepatopancreas in the group reared at 28 °C without ammonia exposure (Fig. 4, L1) showed normal parenchyma with abundant glycogen in the cytoplasm of hepatocytes and intact pancreatic acini. However, in the fish group reared at 32 °C without ammonia exposure (Fig. 3, L2), and those exposed to sub-lethal ammonia and reared at 28 °C and 32 °C (Fig. 3, L3-L4), there was a loss of cell boundaries and a decrease in the glycogen content in the cytoplasm of the hepatocytes. Moreover, there was a dilatation and congestion of the blood vessels with necrosis of the pancreatic acinar cells.

#### Posterior kidney

The posterior kidney in the group reared at 28 °C without ammonia exposure (Fig. 4, K1) showed normal parenchyma with slight interstitial infiltration of the inflammatory cells. However, the fish group reared at 32 °C without ammonia exposure (Fig. 3, K2), and those exposed to sub-lethal ammonia and reared at 28 °C and 32 °C (Fig. 3, K3-K4), triggered necrosis of renal tubules with infiltration of inflammatory cells, dilatation of the bowman’s space with congestion of the blood vessels, and extravasation of blood cells within the interstitial spaces. It was worth noting that the severity of the renal damage was increased along with the elevation of water temperature under sub-lethal ammonia exposure.

### Haematological picture

The changes occurred in the hematological picture of fish that were exposed to different ammonia levels (0 and 10 mg/L) and reared at different water temperatures (28 and 32 °C) for 28 days are demonstrated in Table 1. RBCs, hemoglobin, hematocrit, and WBCs values were significantly impacted by changed temperature, ammonia stress, and their interaction (*P* < 0.05). RBCs, hemoglobin, and hematocrit values were significantly (*P* < 0.05) decreased by increasing temperature, and at the same temperature, they were further significantly (*P* < 0.05) decreased by ammonia stress (Table 1). WBCs were significantly (*P* < 0.05) decreased under ammonia stress, while significantly increased with increasing temperature.

**Table 1:** The effects of different TAN levels and temperatures on the hematological picture of *P. hypophthalmus*.

Temperatures	TAN (mg/L)	RBCs (× 10^6^ µL)	Hemoglobin (g/dL)	Haematocrit (%)	WBCs (× 10^3^ µL)
28 °C	0	2.37 ± 0.03 ^a^	12.65 ± 0.04 ^a^	26.20 ± 0.20 ^a^	20.39 ± 0.62 ^c^
	10	1.77 ± 0.04 ^c^	9.30 ± 0.10 ^b^	20.21 ± 0.31 ^b^	15.95 ± 0.84 ^d^
32 °C	0	2.02 ± 0.07 ^b^	9.27 ± 0.05 ^b^	19.71 ± 0.18 ^b^	39.68 ± 0.51 ^a^
	10	1.41 ± 0.07 ^d^	8.08 ± 0.15 ^c^	13.39 ± 0.61 ^c^	32.87 ± 0.78 ^b^
Two-way ANOVA		P values			
Temperature		< 0.0001	< 0.0001	< 0.0001	< 0.0001
Ammonia		< 0.0001	< 0.0001	< 0.0001	< 0.0001
Temperature ⋅ Ammonia		0.002	< 0.0001	0.032	0.013

Means having different superscript letters in the same column are significantly different at p < 0.05 (Tukey’s HSD test).

### Stress indicators and liver function enzymes

The changes that occurred in the stress indicators (serum cortisol and blood glucose) and liver function enzymes (ALT, AST, and ALP) of fish that were exposed to different ammonia levels (0 and 10 mg/L) and reared at different water temperatures (28 and 32 °C) for 28 days are illustrated in Table 2. It was noticed that serum cortisol, glucose, ALT, ALP, and AST values were significantly influenced by changes in temperature, ammonia stress, and their interaction (*P* < 0.05; Table 2). All the above-mentioned parameters were significantly increased by elevated temperature and further significantly increased under ammonia stress (*P* < 0.05). Their highest values were recorded in the fish group exposed to ammonia stress and reared at 32 °C, compared to those reared at 28 °C.

**Table 2:** The effects of different TAN levels and temperatures on stress markers (blood glucose and serum cortisol levels) and liver function enzymes of *P. hypophthalmus*.

Temperature	TAN (mg/L)	Cortisol (ng/dL)	Glucose (mg/dL)	ALP (IU/L)	AST (IU/L)	ALT (IU/L)
28 °C	0	6.85 ± 0.06 ^d^	4.36 ± 0.14 ^d^	1.08 ± 0.04 ^c^	57.49 ± 1.23 ^d^	3.11 ± 0.09 ^c^
	10	17.96 ± 0.24 ^c^	14.74 ± 0.17 ^c^	3.13 ± 0.06 ^b^	77.05 ± 1.22 ^c^	6.83 ± 0.23 ^b^
32 °C	0	24.42 ± 0.21 ^b^	18.30 ± 0.60 ^b^	3.47 ± 0.12 ^b^	90.84 ± 1.94 ^b^	6.69 ± 0.19 ^b^
	10	35.92 ± 0.48 ^a^	19.52 ± 0.22 ^a^	4.69 ± 0.10 ^a^	98.62 ± 1.56 ^a^	7.65 ± 0.26 ^a^
Two-way ANOVA		P values				
Temperature		< 0.0001	< 0.0001	< 0.0001	< 0.0001	< 0.0001
Ammonia		< 0.0001	< 0.0001	< 0.0001	< 0.0001	< 0.0001
Temperature ⋅ Ammonia		0.001	< 0.0001	0.001	0.005	< 0.0001

Means having different superscript letters in the same column are significantly different at p < 0.05 (Tukey’s HSD test).Alkaline phosphatase (ALP); Alanine aminotransferase (ALT), Aspartate Aminotransferase (AST).

### Lipid peroxidation and oxidative stress biomarkers

The changes that occurred in the lipid peroxidation indicator (MDA) and oxidative stress biomarkers (CAT, SOD, GPx, and GST) in the liver of fish that were exposed to different ammonia levels (0 and 10 mg/L) and reared at different water temperatures (28 and 32 °C) for 28 days are summarized in Table 3. It was observed that MDA, CAT, SOD, GPx, and GST values were significantly affected by elevated temperature, ammonia exposure, and their interaction. Ammonia exposure significantly increased their values, and these values continued to increase with elevated temperature, reaching their highest levels in the ammonia-stressed fish group reared at 32 °C compared to those reared at 28 °C without ammonia exposure.

**Table 3:** The effects of different TAN levels and temperatures on the lipid peroxidation indicator and oxidative stress biomarkers of *P. hypophthalmus*.

Temperature	TAN (mg/L)	MDA (nmol/mg protein)	CAT (U/mg protein)	GST (U/mg protein)	SOD (U/mg protein)	GPx (U/mg protein)
28 °C	0	11.84 ± 0.38 ^c^	23.14 ± 0.58 ^c^	19.28 ± 0.49 ^d^	2.55 ± 0.08 ^d^	17.35 ± 0.44 ^c^
	10	39.53 ± 0.64 ^b^	30.25 ± 0.48 ^b^	25.62 ± 1.00 ^c^	3.88 ± 0.05 ^c^	19.62 ± 0.85 ^c^
32 °C	0	38.11 ± 1.23 ^b^	33.69 ± 0.85 ^b^	31.89 ± 0.80 ^b^	5.36 ± 0.16 ^b^	23.60 ± 0.59 ^b^
	10	52.34 ± 0.85 ^a^	44.05 ± 0.71 ^a^	42.37 ± 1.66 ^a^	8.16 ± 0.11 ^a^	28.44 ± 1.23 ^a^
Two-way ANOVA		P values				
Temperature		< 0.0001	< 0.0001	< 0.0001	< 0.0001	< 0.0001
Ammonia		< 0.0001	< 0.0001	< 0.0001	< 0.0001	0.003
Temperature ⋅ Ammonia		< 0.0001	0.042	0.001	< 0.0001	0.0160

Means having different superscript letters in the same column are significantly different at p < 0.05 (Tukey’s HSD test).Malondialdehyde (MDA; lipid peroxidation biomarker); Catalase enzyme (CAT), Glutathione-S-transferase (GST); superoxide dismutase (SOD); glutathione peroxidase enzyme (GPx)

## Discussion

In this study, fish exposure to elevated ammonia levels resulted in a range of clinical signs, with the specific presentation varying across species. Herein, there were behavioral alterations, sloughing at caudal peduncle, dropsy, and dermal ulcerations of the exposed *P. hypophthalmus*. The gills may show signs of congestion and hemorrhage, consistent with observations in other species^30^. In goldfish, clinical signs include hyperexcitability, hyperventilation, and gill congestion and hemorrhage^31^. Similar findings have been reported in yellow catfish, which showed increased respiratory movements, hyperexcitability, and a greyish skin color^32^. The pathological changes observed in the gills are a key feature of ammonia toxicity. Elevated ammonia concentrations can trigger hyperplasia of the gill filaments, which is a protective mechanism that helps shield the delicate gill tissues from the toxic effects of ammonia. This response can, however, lead to secondary issues such as hyperventilation and gill congestion^4^.

Studying the histopathology of fish is crucial practice in aquaculture because it helps us determine how environmental stressors and aquatic pollutants affect the tissues of exposed fish^33–35^. This method allows researchers to visually assess and analyze the microscopic changes in fish organs and tissues, providing valuable insights into their overall health and well-being. By examining these changes, we can better understand the negative impacts of pollution and other stressors, ultimately leading to improved management and conservation strategies in aquatic environments. The gills, serving as the main point of contact between the fish and its aqueous environment, are crucial for several physiological processes including gas exchange, osmoregulation, and the excretion of nitrogenous wastes^36^. Because of this direct exposure, the histological structure of gill tissues can serve as a sensitive bioindicator of water quality, with changes often reflecting exposure to pollutants^37^. Similarly, the liver plays a central role in the detoxification of harmful substances, and consequently, histological alterations in hepatic tissues are commonly observed in fish exposed to aquatic toxicants and other stressors^38^. Exposure to pollutants and toxicants impacts the renal tissues of fish, with the posterior kidneys being particularly vulnerable^39^. Fish muscle development, or myogenesis, at any life stage, is a result of the equilibrium between muscle fiber hypertrophy (the growth of existing muscle fibers) and hyperplasia (the formation of new muscle fibers)^40^. This process, along with overall muscle cellularity and the resulting flesh quality, is typically influenced by a range of intrinsic (e.g., genetic makeup) and extrinsic (e.g., environmental conditions) factors^41,42^. Fish livers play a crucial role in digestion, detoxification, and the biotransformation of harmful substances^38^. Consequently, they’re considered a dependable biomarker and a key indicator of the overall health of fish^43^. This study revealed that ammonia exposure at higher temperatures caused severe histopathological alterations in gills, musculature, posterior kidney and liver compared to those exposed to ammonia at lower temperatures. Esam et al^18^. also elucidated that high water temperature aggravated the histopathological lesions in gills and hepatic tissues of Nile tilapia exposed to sub-lethal ammonia exposure. The gill is a vital organ for a fish’s survival, functioning similarly to a lung for terrestrial animals. It’s responsible for gas exchange, allowing the fish to extract dissolved oxygen from the water. Within the gills are specialized cells, known as chloride cells, which play a crucial role in maintaining the fish’s osmotic balance (the regulation of water and salt concentrations inside the body). According to Abdel-Latif et al^22^., when fish are continuously exposed to toxins and contaminants in their aquatic environment, these delicate structures can be significantly damaged. This damage affects the chloride cells’ ability to regulate ions and the gill tissues’ overall integrity.

Analysis of fish blood serves as a rapid clinical diagnostic instrument in aquaculture research, providing insight into the overall physiological state of fish. It helps in understanding their nutritional condition, well-being, and health status^44^. In the present study, RBCs count, Hb, and Htc significantly decreased with the increase in water temperature under ammonia exposure. However, WBCs increased significantly with the increase in water temperature to 32 °C compared to those reared at 28 °C. This increase may be attributed to the immune system response of fish to counteract the negative effects of stressors exerted on the exposed fish. Shin et al^11^. found that RBCs count, WBC count, Hb, and Htc values significantly decreased in *S. schlegelii* by ammonia exposure at high water temperatures. Indeed, the hematological indices are crucial diagnostic tools for evaluating the health and immune status of fish. Both red and white blood cell indices are commonly utilized for this purpose^45^. Deficiencies in red cell indices have been linked to blood disorders like anemia, polycythemia, and iron deficiency. The role of white cell indices, on the other hand, is associated with the body’s immune system and its phagocytic responses^46–48^.

ALT, AST, and ALP were significantly increased in the ammonia-exposed fish group reared at higher temperature at 32 °C compared to those reared at 28 °C. In the same sense, Shin et al^11^. reported that elevation of water temperature led to alterations of the hepatic enzymes in *S. schlegelii* exposed to ammonia stress. Hepatic enzymes like AST, ALP, and ALT typically exist in low concentrations in a healthy fish’s bloodstream. However, when liver cells are damaged by stressors, these enzymes may abnormally leak into the blood, causing their levels to rise^49^. Serum cortisol levels were significantly increased in ammonia-exposed fish group reared at higher temperatures. Blood glucose and cortisol levels were also increased in *A. fimbria* that were exposed to ammonia and reared at high water temperatures than those reared at low water temperatures^50,51^. It is well-known that stressful conditions in fish lead to increased cortisol secretion, making elevated blood cortisol a reliable bio-indicator of stress exposure^52,53^. To provide the energy needed to mitigate with these stressors, fish also commonly secrete blood glucose^54^. Moreover, the hyperglycemia that occurred in the exposed fish may be associated with the glycogenolytic activity of catecholamines and gluconeogenetic effects of glucocorticoids that are released by the stress response^53^.

When fish are exposed to environmental stressors, their bodies may struggle to manage the balance between oxidants and antioxidants, a condition known as oxidative stress^55^. This imbalance is caused by an uncontrolled generation of free radicals and reactive oxygen species (ROS)^56^. The resulting surge of these molecules can harm cells in various ways, from damaging lipids and proteins to causing DNA damage, which can then trigger programmed cell death (apoptosis)^57^. In fish, this is often combated by a suite of endogenous enzymatic antioxidants such as SOD, CAT, and GPx, which work to neutralize these reactive species^57^. When these protective mechanisms are compromised or the production of free radicals is excessive, it can result in oxidative damage. A key consequence of oxidative stress is lipid peroxidation, a process that damages cell membranes and produces byproducts like MDA. Consequently, the levels of antioxidant enzymes (SOD, CAT, and GPx) and the concentration of damage markers (MDA) are often measured to assess the degree of oxidative stress^58^. These markers are considered reliable bioindicators of an organism’s physiological response to environmental stressors. Ammonia exposure and heat stress are the main factors in inducing oxidative stress in fish^18^. Herein, it was noticed that MDA, CAT, SOD, GPx, and GST values significantly increased in the ammonia-exposed fish group reared at higher temperature and their highest values were recorded in the fish group exposed to sub-lethal ammonia and reared at 32 °C compared to those reared at 28 °C. The observed elevation in antioxidant enzymes (CAT, SOD, and GPx) reflects a compensatory physiological response to increased ROS generation under ammonia and thermal stress. However, the concurrent increase in MDA levels indicates that antioxidant defenses were insufficient to fully counteract oxidative damage, suggesting a state of oxidative imbalance. These effects may have resulted from the increased and excessive ROS generation^59^. It is observed that the increased CAT, SOD, GPx, and GST levels may be associated with the normal antioxidant defense mechanisms that occur in the fish body to mitigate oxidative stress responses^57^, that resulted from fish exposure to ammonia and/or high-water temperatures. Similar findings were found in several studies. For example, it was found that ammonia exposure at higher temperatures caused a significant elevation of the antioxidant defence enzymes (SOD and CAT) in *A. fimbria*^50,51^. Recently, it was found that long-term ammonia and heat stress increased CAT and GPx enzyme activities as well as MDA levels in rice field eel juveniles^60^. Indeed, temperature is a crucial factor that influences oxidative stress as the rise in water temperatures will consequently lead to an increase of oxygen consumption and promoting ROS generation^61^. Despite the strengths of this study, some limitations should be acknowledged, including the relatively short experimental duration, controlled laboratory conditions, and limited sample size.

## Conclusions

This study addresses a practically relevant aquaculture challenge under Egyptian conditions, where ammonia accumulation and thermal fluctuations commonly threaten pangasius production. In short, ammonia exposure at elevated water temperatures can induce considerable clinical and histopathological lesions, exaggerated stress, and alterations in the antioxidant responses. The study demonstrates that ammonia exposure in *P. hypophthalmus* is significantly exacerbated by elevated ambient temperatures. The synergistic effect of 10 mg/L TAN and a 32 °C environment triggers a cascade of physiological failures, ranging from severe tissue necrosis and respiratory distress in the gills to systemic oxidative stress and suppressed hematological profiles. These findings confirm that higher temperatures reduce the fish’ threshold for ammonia tolerance, transforming sub-lethal concentrations into potent stressors that compromise both physical integrity and metabolic homeostasis. As global water temperatures continue to rise due to climate change, these results highlight the urgent need for stricter water quality management in tropical aquaculture.

## Data Availability

The datasets used and/or analyzed during the current study available from the corresponding author on reasonable request.

## Declarations

### ARRIVE guidelines

All methods used in the present research were carried out in accordance with ARRIVE guidelines.
